# Tumor Cell-Derived Exosomal miR-770 Inhibits M2 Macrophage Polarization *via* Targeting MAP3K1 to Inhibit the Invasion of Non-small Cell Lung Cancer Cells

**DOI:** 10.3389/fcell.2021.679658

**Published:** 2021-06-14

**Authors:** Jixian Liu, Ruixing Luo, Junbin Wang, Xinyu Luan, Da Wu, Hua Chen, Qinghua Hou, Guangxian Mao, Xiaoqiang Li

**Affiliations:** Department of Thoracic Surgery, Peking University Shenzhen Hospital, Shenzhen, China

**Keywords:** NSCLC, miR-770, MAP3K1, exosome, macrophages

## Abstract

**Background:**

Non-small cell lung carcinoma (NSCLC) is a type lung cancer with high malignant behaviors. MicroRNAs (miRNAs) are known to be involved in progression of NSCLC. In order to explore potential targets for the treatment of NSCLC, bioinformatics tool was used to analyze differential expressed miRNAs between NSCLC and adjacent normal tissues.

**Methods:**

Bioinformatics tool was used to find potential targets for NSCLC. Cell proliferation was investigated by Ki67 staining. Cell apoptosis was measured by flow cytometry. mRNA and protein expression in NSCLC cells were detected by RT-qPCR and Western-blot, respectively. Transwell assay was performed to test the cell migration and invasion. In order to investigate the function of exosomal miRNA in NSCLC, *in vivo* model of NSCLC was constructed.

**Results:**

MiR-770 was identified to be downregulated in NSCLC, and miR-770 agomir could significantly inhibit NSCLC cell proliferation through inducing the apoptosis. Additionally, the metastasis of NSCLC cells was decreased by miR-770 agomir. MAP3K1 was identified to be the target mRNA of miR-770. Meanwhile, tumor cell-derived exosomal miR-770 inhibited M2 macrophage polarization *via* downregulation of MAP3K1, which in turn suppressed NSCLC cell invasion. Besides, tumor cell-derived exosomal miR-770 markedly decreased NSCLC tumor growth *in vivo* through suppressing M2 macrophage polarization.

**Conclusion:**

Tumor cell-derived exosomal miR-770 inhibits M2 macrophage polarization to inhibit the invasion of NSCLC cells *via* targeting MAP3K1. Thus, this study provided a new strategy for the treatment of NSCLC.

## Introduction

Lung cancer is the most frequently diagnosed malignant tumor globally (Nelson et al., 2020). Meanwhile, lung malignant tumors are classified into small cell lung cancer and non-small cell lung cancer (NSCLC) (Abedi Gaballu et al., 2019). NSCLC accounts for over 80% of lung cancers, and about 80% of patients are found usually in the advanced stages of NSCLC (Chen Y. et al., 2020). Great attentions have been paid to treat NSCLC, while the effect is still not ideal (Iwama et al., 2020). Thereby, it is necessary to find new methods for NSCLC treatment.

MicroRNAs (miRNAs) are endogenous RNAs featured by a closed cyclic structure (Wei et al., 2021). In addition, miRNAs are known to exert their biological functions *via* regulation of the target mRNAs (Guo et al., 2020). This greatly modulates the cellular process, including cell growth (Bengone-Abogourin et al., 2019; Torma et al., 2019). Meanwhile, miRNAs are involved in NSCLC progression. For instance, He (2017) indicated that downregulation of miR-15a could promote the development and epithelial-mesenchymal transition (EMT) process of NSCLC cells; Xiong et al. (2018) revealed that miR-9 could attenuate the symptom of NSCLC. Therefore, miRNAs can play important roles in NSCLC. However, the functions of miRNAs in NSCLC need to be further explored.

Previous reports have revealed that the association between tumor cells and stroma cells in tumor microenvironment can play a key role in modulating the development of cancer (Maia et al., 2020; Zeng and Fu, 2020). In tumor environment, macrophages are often associated with tumor cells to regulate tumorigenesis and metastasis of cancers (Li et al., 2020b; Wang and Ilyas, 2020). In addition, the macrophages of the tumor environment showing oncogenic effects are considered as tumor-associated macrophages (TAMs), which usually exhibit M2 phenotype (Dessouki et al., 2020). The correlation between tumor cells and TAMs has been revealed some cancers (gastric cancer, pancreatic cancer, et al.) (Gao et al., 2020; Zhou et al., 2021). Nevertheless, the detailed association between NSCLC treatments with TAMs is unclear.

In this study, we sought to investigate the differentially expressed miRNAs which are associated with the development of NSCLC. We hope this research would shed new lights on exploring the new methods for NSCLC treatment.

## Materials and Methods

### Cell Culture

Non-small cell lung carcinoma cell lines (SK-MES-1, A549, and NCI-H1650), BEAS-2B and THP-1 cells were purchased from ATCC (Manassas, VA, United States) and maintained in DMEM (Thermo Fisher Scientific, Waltham, MA, United States) containing 10% FBS, 1% streptomycin and penicillin (Thermo Fisher Scientific) in an incubator (37°C, 5% CO_2_).

### Reagents

IL-4 and IL-13 were obtained from MedChemExpress (MCE, Monmouth Junction, NJ, United States).

### Bioinformatics Analysis

The Cancer Genome Atlas Program (TCGA) dataset^1^ was used to explore the differentially expressed miRNAs between adjacent normal tissues and NSCLC tissues. The analysis was performed according to the prognosis information of lung adenocarcinoma (LUAD) and lung squamous cell carcinoma (LUSC) in TCGA. Meanwhile, LUAD and LUSC are the two main subtypes of NSCLC. In addition, TCGA dataset was downloaded and R analysis was performed as previously described (Colaprico et al., 2016).

### Cell Transfection

Non-small cell lung carcinoma cells were transfected with miR-770 agomir or agomir-ctrl (NC; GenePharma, Shanghai, China) by using Lipofectamine 2000 (Thermo Fischer Scientific).

### Isolation and Identification of Exosome

After 48 h of culture, NSCLC cell supernatants were collected by centrifugation (300 × *g* for 15 min, 2000 × *g* for 15 min, and 10,000 × *g* for 30 min). Subsequently, cell supernatants were filtrated and collected to isolate exosomes *via* ultracentrifugation (120,000 × *g* for 70 min). The particle sizes of exosomes were investigated by Particle Metrix (PMX), the structure of exosomes was observed by transmission electron microscopy (TEM) and the exosome markers were detected by western blot (Madhyastha et al., 2021; Xing et al., 2021).

### Isolation and Cultivation of Macrophages

Macrophages were treated with PMA (100 ng/ml; Peprotech), and then treated with PBS, A549-Exo-NC, A549-Exo-miR-770 agomir or 20 ng/ml IL4/IL-13 for 24 h. Subsequently, the macrophages surface maker CD206 (BD Biosciences, New Jersey, United States) and CD86 (BD Biosciences) were analyzed by flow cytometry (BD Biosciences).

### Reverse Transcription-Quantitative PCR

TRIzol^®^ reagent (Takara, Tokyo, Japan) Was used to isolate total RNA From cell lines or tissues. the PrimeScript RT reagent kit (ELK bioscience, Wuhan, China) Was used to reverse transcribe total RNA Into cDNA. Subsequently, the SYBR premix Ex Taq II kit (ELK bioscience) was used in RT-qPCR. the protocol Was as follows: 2 min at 94°C, followed by 35 cycles (30 s at 94°C and 45 s at 55°C). the primer sequences Were listed as follows: miR-770 forward, 5′-CTCGCTTCGGCAGCACAT-3′ and reverse, 5′-AACGCTTCACGAATTTGCGT-3′; Arginase-1 forward 5′-AGACCACAGTTTGGCAATTGG-3′ and reverse, 5′-AGGAGAATCCTGGCACATCG-3′; iNOS forward, 5′-GCAG GACTCACAGCCTTTGG-3′ and reverse, 5′-GGCTGGATGT CGGACTTTGT-3′; β-actin forward, 5′-GTCCACCGCAAATG CTTCTA-3′ and reverse, 5′-TGCTGTCACCTTCACCGTTC-3′; MAP3K1 forward 5′-AGCCACGAGTTGTCAAGTCCT-3′ and reverse, 5′-AGCAGGAGGGATTTGCTGAG-3′; And U6 forward, 5′-GTCCACCGCAAATGCTTCTA-3′ and reverse, 5′-TGCTGTCACCTTCACCGTTC-3′. 2^–Δ^ ^Δ^ ^*t*^ method Was used for quantification. β-actin or U6 Was used as a loading control.

### CCK-8 Assay

Non-small cell lung carcinoma cells (5 × 10^3^ cells/well) were seeded overnight. Then, cells were treated with agomir-ctrl or miR-770 agomir at 37°C for 0, 24, 48 or 72 h. Subsequently, cells were added with CCK-8 reagents (10 μl; Beyotime) at 37°C for 2 h. The absorbance (450 nm) was measured by a microplate reader.

### Cell Apoptosis Detection

Non-small cell lung carcinoma cells (5 × 10^4^) were centrifuged (956 *g*, 5 min) and then the residue was resuspended. Subsequently, cells were treated with 5 μl Annexin V-FITC and propidium iodide (PI) in the dark at 4°C for 15 min. Flow cytometer (BD) was used to analyze the cell apoptosis. The data were quantified by FlowJo (BD).

### Western Blotting

RIPA lysis buffer (Beyotime) was used to isolate total protein from cells. BCA kit (Beyotime) was used to quantify total protein. SDS-PAGE (10%) was used to separate protein (40 μg per lane), and then proteins were transferred onto PVDF membranes (Thermo Fisher Scientific). After blocked with 5% skimmed milk for 1 h, membranes were incubated with primary antibodies overnight as follows: anti-CD63 (Abcam; 1:1,000), anti-TSG101 (Abcam; 1:1,000), anti-CD9 (Abcam; 1:1,000), anti-CD81 (Abcam; 1:1,000), anti-MAP3K1 (Abcam; 1:1,000), anti-p-JNK (Abcam; 1:1,000), anti-JNK (Abcam; 1:1,000), anti-E-cadherin (Abcam; 1:1,000), anti-N-cadherin, anti-vimentin (Abcam; 1:1,000), anti-ERK1/2 (Abcam; 1:1,000), anti-p-ERK1/2 (Abcam; 1:1,000) and anti-β-actin (Abcam; 1:1,000). After that, the membranes were incubated with secondary antibodies (HRP-conjugated, Abcam; 1:5,000) for 1 h. ECL kit (Thermo Fisher Scientific) was used to visualize protein bands. β-actin was used for normalization.

### Immunofluorescence

Non-small cell lung carcinoma cells were fixed in methanol for 20 min. Then, cells were incubated with anti-Ki67 (1:1,000; Abcam), anti-CD63 (1:1,000; Abcam) or anti-PKH26 (1:1,000; Abcam). After that, cells were incubated with the secondary antibody (1:5,000; goat anti-rabbit IgG, Abcam). Finally, the result was observed by a microscope (Olympus, Tokyo, Japan).

### Dual Luciferase Reporter Assay

MAP3K1 containing the putative binding sites of miR-770 was obtained from Genepharma and cloned into the vectors (pGL6; Beyotime) for establishment of reporter vectors MAP3K1 (WT/MT). MAP3K1 (WT/MT) was transfected into NSCLC cells together with vector-control (NC) or miR-770 agomir using Lipofectamine 2000 (Thermo Fisher Scientific). The result was analyzed by the Dual-Glo Luciferase Assay System (Promega).

### Transwell Assay

The upper chamber was pre-treated with 100 μl Matrigel (the migration assay did not include the Matrigel). Subsequently, NSCLC cells (1.0 × 10^6^) were plated into the upper chamber in RPMI1640 medium containing 1% FBS. Meanwhile, the lower chamber was treated with RPMI1640 medium containing 10% FBS. After 24 h of incubation, cells in the lower chamber were stained with 0.1% crystal violet, and then the result was observed and counted by a light microscope.

### RNA Pull-Down

For the RNA pulldown assay, the Biotin RNA Labeling Mix (Roche, Basel, Switzerland) was used to transcribe and label probe-control or probe-miR-770 *in vitro*. An RNA structure buffer (Thermo, MA, United States) was used to induce secondary structure formation from the biotin-labeled RNAs. The biotinylated miR-770 and negative control (bio-NC) were generated *via* GenePharma and coated to streptavidin-conjugated magnetic beads. Cells were lysed and then incubated with the magnetic beads for 6 h. The RNA on the beads was isolated and the enrichment level of MAP3K1 was detected by PCR.

### Enzyme Linked Immunosorbent Assay

The levels of IL-10 and TGF-β in supernatants of NSCLC cells were assessed by ELISA kit [Multisciences (Lianke) Biotech, Hangzhou, China].

### Immunohistochemical Staining

Tissues of mice were fixed overnight, and then cut into thick sections (5 μm). The sections were deparaffinized and rehydrated. For antigen retrieval, sections were heated in a microwave (with sodium citrate buffer). Subsequently, samples were washed with PBS for 5 min. Then, samples were incubated in 3% H_2_O_2_ for 25 min, washed and incubated in serum for 30 min. After that, the samples were incubated with anti-Ki-67 (Abcam) or anti-CD206 (Abcam) at 4°C, followed with secondary antibody (HRP-labeled; Abcam) for 30 min at 37°C. Finally, diaminobenzidine (DAB) was added and the tissues were observed under a microscope. All the antibodies were obtained from Abcam.

### *In vivo* Study

BALB/c nude mice (n = 24; 6–8 weeks old) were obtained from Vital River (Beijing, China). The protocols for animal care and use of laboratory animals were in accordance with ethical committee of Peking University Shenzhen Hospital. A549 cells co-cultured with macrophages, macrophages-exo, macrophages^*exo–NC*^ or macrophages^*exo–miR–770 agomir*^ were subcutaneously transplanted in mice. The tumor volume was investigated once a week as follow: length × width × width. In the end, mice were sacrificed for tumor tissue collection. All *in vivo* experiments were performed in accordance with National Institutes of Health guide for the care and use of laboratory animals.

### TUNEL Staining

Briefly, paraffin sections were washed, permeabilized, and then incubated with 50 μl TUNEL reaction mixtures in a wet box for 60 min at 37°C in the dark. For signal conversion, slides were incubated with 50 μl of peroxidase (POD) for 30 min at 37°C, rinsed with PBS, and then incubated with 50 μl diaminobenzidine (DAB) substrate solution for 10 min at 25°C. Finally, the tissues were observed under an optical microscope.

### Statistical Analysis

Data are presented as the mean ± standard deviation. CCK8 assay was performed in quintuplicate. Flow cytometry, western blot, RT-qPCR and ELISA were repeated in triplicate. The other experiments were performed in three times. In addition, Comparisons between two groups were analyzed by the unpaired student’s *t*-test. All other experiments were repeated three times. One-way analysis of variance and Tukey’s *post hoc* tests were used for comparisons between ≥ 3 groups. *P* < 0.05 was considered to indicate a statistically significant difference.

## Results

### Differentially Expressed miRNAs in NSCLC

**FIGURE 1:**
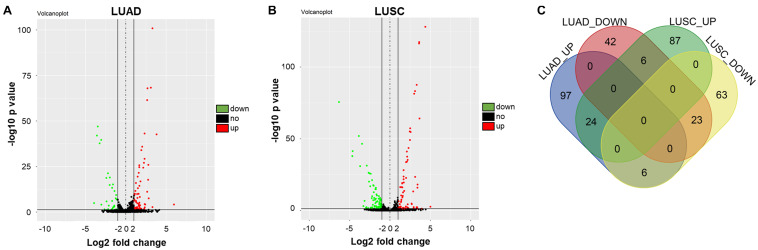
Differentially expressed miRNAs in NSCLC. **(A,B)** Volcano plots illustrating the miRNAs differentially expressed in NSCLC detected in the TCGA dataset. Red indicates a higher expression level, while blue indicates a lower expression level. **(C)** Venn diagram showing the overlap among the differentially expressed miRNAs in TCGA dataset. Among these differentially expressed miRNAs in TCGA, 23 were commonly downregulated, whereas 24 were commonly upregulated.

To detect differentially expressed miRNAs in NSCLC, bioinformatics analysis was performed. As illustrated in Figures 1A,B, the differentially expressed miRNAs in NSCLS tissues compared to adjacent normal tissues in TCGA dataset were presented as volcano plots. Overlap among these datasets was indicated by the Venn diagram in Figure 1C. Among these differentially expressed miRNAs in TCGA, 23 were commonly downregulated, whereas 24 were commonly upregulated. Moreover, among the overlap of these differentially expressed miRNAs, miR-770 was closely associated with the tumorigenesis of NSCLC (Zhang et al., 2017). Therefore, miR-770 was selected for further analysis.

### MiR-770 Agomir Significantly Inhibited the Proliferation of NSCLC Cells *via* Inducing Apoptosis

**FIGURE 2:**
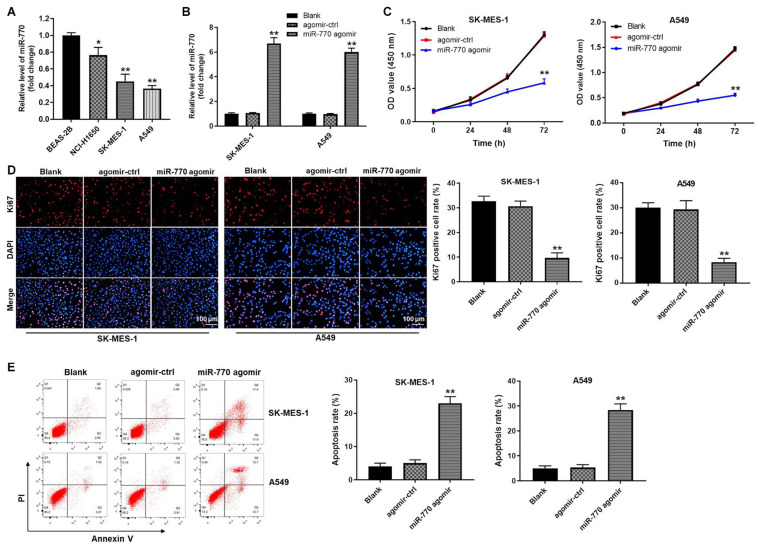
MiR-770 agomir significantly inhibited the proliferation of NSCLC cells *via* inducing apoptosis. **(A)** The level of miR-770 in BEAS-2B, NCI-H1650, SK-MES-1 or A549 cells was detected by RT-qPCR. **(B)** SK-MES-1 or A549 cells were transfected with agomir-ctrl or miR-770 agomir. Then, the expression of miR-770 in NSCLC cells was tested by RT-qPCR. **(C)** The viability of NSCLC cells was tested by CCK-8 assay. **(D)** The proliferation of NSCLC cells was investigated by Ki67 staining. **(E)** The apoptosis of NSCLC cells was tested by flow cytometry. *N* = 3; ^∗∗^*P* < 0.05 compared to control.

In order to investigate the role of miR-770 in NSCLC cells, RT-qPCR was performed. As revealed in Figure 2A, the expression of miR-770 was significantly downregulated in A549, SK-MES-1 and NCI-H1650 cells, compared with that in BEAS-2B cells. Meanwhile, the level of miR-770 was markedly upregulated in A549 and SK-MES-1 cells transfected with miR-770 agomir (Figure 2B). In addition, miR-770 overexpression notably decreased the viability and proliferation of NSCLC cells (Figures 2C,D, Supplementary Figures 1A,B). Moreover, miR-770 agomir significantly induced the apoptosis of NSCLC cells (Figure 2E and Supplementary Figure 1C). Taken together, miR-770 agomir could inhibit the proliferation of NSCLC cells *via* inducing apoptosis.

### MiR-770 Agomir Inhibited the Migration and Invasion of NSCLC Cells

**FIGURE 3:**
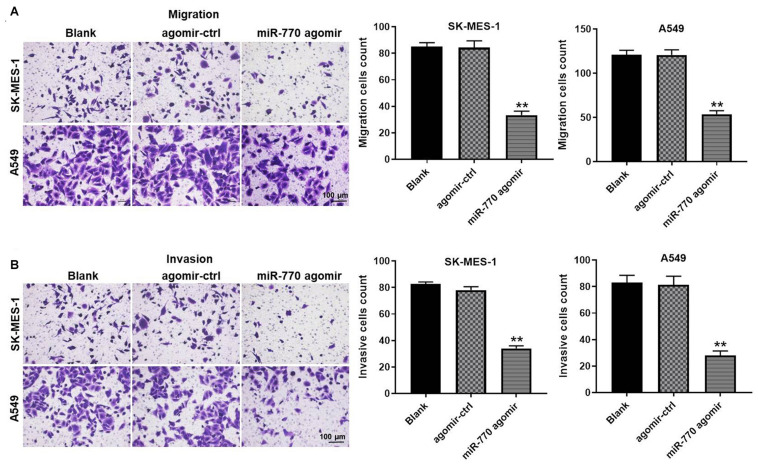
MiR-770 agomir inhibited the migration and invasion of NSCLC cells. **(A)** The migration of NSCLC cells was investigated by transwell assay. **(B)** The invasion of NSCLC cells was investigated by transwell assay. *N* = 3; ^∗∗^*P* < 0.05 compared to control.

In order to detect the effect of miR-770 on NSCLC cell migration and invasion, transwell assays were used. As revealed in Figure 3A, overexpression of miR-770 obviously decreased the migration of NSCLC cells. Consistently, the cell invasion was notably inhibited in the presence of miR-770 agomir (Figure 3B). In summary, miR-770 agomir could inhibit the migration and invasion of NSCLC cells.

### MiR-770 Can Be Transferred From A549 Cells to Macrophages Cells *via* Exosomes

**FIGURE 4:**
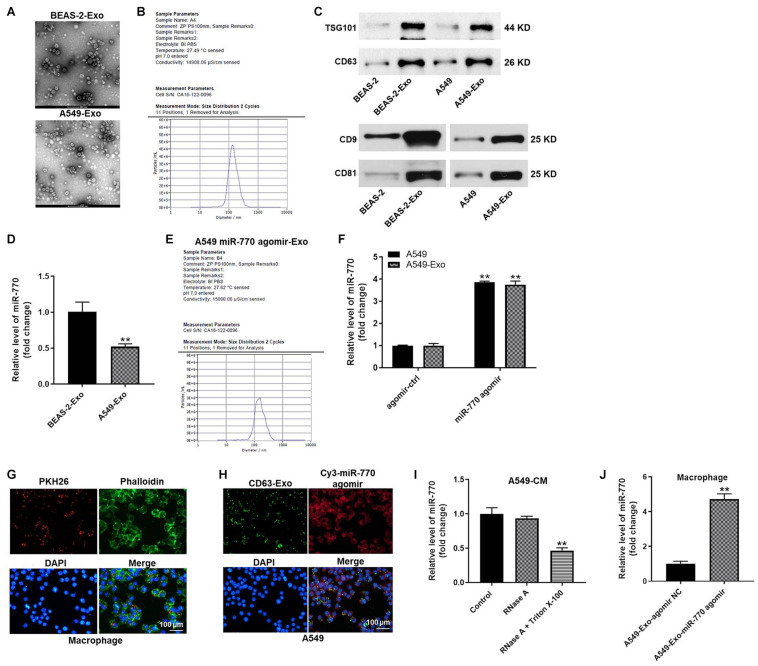
Exosomes were successfully isolated from NSCLC cells. **(A)** The separation efficiency of exosomes was examined by TEM. **(B)** The particle sizes of exosomes derived from BEAS-2B (BEAS-2B-exo) were measured by NTA. **(C)** The expressions of TSG101, CD63, CD9 and CD81 in BEAS-2B, A549 cells, BEAS-2B-exo or A549-exo were detected by western blot. **(D)** The expression of miR-770 in BEAS-2B-exo or A549-exo was investigated by RT-qPCR. **(E)** The particle sizes of exosomal miR-770 derived from A549 cells were measured by NTA. **(F)** The level of miR-770 in NSCLC cells or NSCLC cell-derived exosomes was investigated by RT-qPCR. **(G)** THP-1 cells were treated with 100 ng/ml PMA, and then co-cultured with A549 cell-derived exosomes. Then, the location of exosomes was observed by immunofluorescence staining. **(H)** GFP-CD63 and fluorescence labeled miR-770 agomir were co-transfected into NSCLC cells. Then, exosomes were isolated and added into macrophages. The location of exosomes was observed by immunofluorescence staining. **(I)** The level of miR-770 in CM of macrophages/miR-770 treated with RNase alone or RNase plus Triton X-100 was detected by RT-qPCR. **(J)** The level of miR-770 in macrophages was detected by RT-qPCR. *N* = 3; ^∗∗^*P* < 0.05 compared to control.

It has been reported that tumor-derived exosomes play important roles in tumorigenesis (Rezaei et al., 2021; Xie et al., 2021). Thus, we sought to isolate the exosomes from A549 cells, and then exosomes isolation was detected by TEM. As shown in Figures 4A,B, typical rounded particles ranging from 30 to 150 nm in diameter was observed by TEM, and Nanoparticle Tracking Analysis (NTA) exhibited a similar size distribution of exosomes. Moreover, the expressions of exosomal proteins (CD63, TSG101, CD9 and CD81) were significantly higher in exosomes derived from NSCLC or BEAS-2B cells (A549-exo and BEAS-2-exo), compared with A549 or BEAS-2B cells (Figure 4C). Meanwhile, the expression of miR-770 in A549-exo was notably downregulated, compared with that in BEAS-2-exo (Figure 4D), and exosomes secreted from A549 cells that were transfected with miR-770 agomir (A549/miR-770 agomir-exo) were approximately 100 nm in diameter (Figure 4E). Furthermore, the level of miR-770 was upregulated in A549/miR-770 agomir and A549/miR-770 agomir-exo compared to that in A549 cells transfected with agomir-ctrl (A549/agomir-ctrl) and exosomes derived from A549/agomir-ctrl (A549/agomir-ctrl-exo) (Figure 4F).

To investigate whether exosomes can mediate cell-to-cell crosstalk by transmitting miR-770 between A549 cells and macrophages (PMA-differentiated human THP-1 monocytes), A549 cell derived PKH26-labeled exosomes were co-cultured with macrophages. After 48 h of co-culture, PKH26 lipid dye were observed in macrophages, indicating that A549-exo could be transferred to macrophages (Figure 4G). In addition, macrophages were incubated with CD63-labeled exosomes derived from A549 cells that were transfected with Cy3-labeled miR-770 agomir. Both CD63 lipid dye and Cy3 fluorescence were observed in macrophages (Figure 4H). These results indicated that miR-770 is contained in A549-exo and can be transferred to macrophages. Meanwhile, the level of miR-770 in conditioned medium (CM) of macrophages/miR-770 was limitedly affected by RNase A but obviously inhibited when treated with RNase A plus Triton X-100 (Figure 4I). Besides, A549/miR-770 agomir-exo significantly increased the level of miR-770 in macrophages (Figure 4J). Altogether, miR-770 can be transferred from A549 cells to macrophages cells *via* exosomes.

### Exosomal miR-770 Inhibited M2 Polarization in Macrophages

**FIGURE 5:**
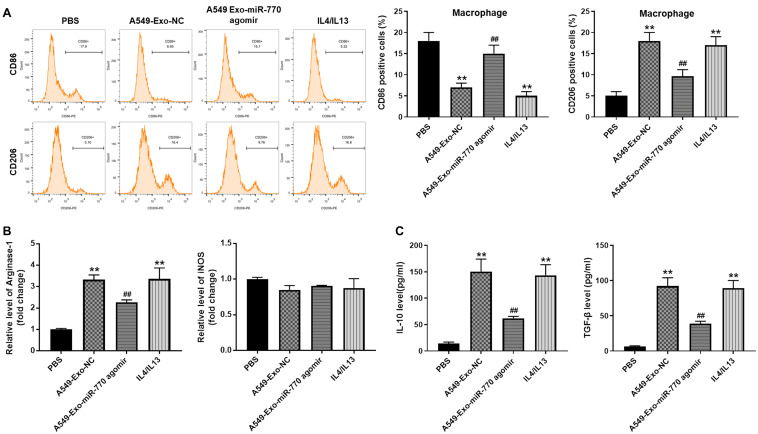
Exosomal miR-770 inhibited M2 polarization in macrophages. **(A)** Macrophages were co-cultured with A549-Exo-NC, A549-Exo-miR-770 agomir or 20 ng/ml IL-4/IL-13 for 24 h. Then, the rate of CD86 or CD206 distribution in macrophages was tested by flow cytometry. **(B)** The levels of Arginase-1 and iNOS in macrophages were tested by RT-qPCR. **(C)** The levels of TGF-β and IL-10 in supernatants of macrophages were detected by ELISA. *N* = 3; **P < 0.01 compared to PBS, ^##^*P* < 0.01 compared to A549-Exo-NC.

In order to detect the role of A549/miR-770 agomir-exo in the polarization of M2 phenotype macrophages, macrophages were treated with 50 μg/ml exosomes derived from A549 cells (IL-4/IL-13 treatment as a positive control), and then flow cytometry was performed. As revealed in Figure 5A, macrophages exhibited a CD86^*low*^/CD206^*high*^ phenotype, when cells were co-cultured with A549/agomir-ctrl-exo; however, when macrophages were incubated with A549/miR-770 agomir-exo, cells exhibited a CD86^*high*^/CD206^*low*^ phenotype (Figure 5A). In addition, macrophages treated with A549/agomir-ctrl-exo expressed notably more M2 representative cytokines Arginase-1, IL-10 and TGF-β, but there were no significant changes of M1 representative cytokine iNOS. In contrast, macrophages incubated with A549/miR-770 agomir-exo showed markedly reduced level of Arginase-1, IL-10 and TGF-β compared to those incubated with A549/agomir-ctrl-exo (Figures 5B,C). These results suggested that exosomes with upregulated miR-770 could inhibit the polarization of M2 macrophages.

### Exosomal miR-770 Significantly Inhibited the Migration, Invasion, and EMT Process of NSCLC Cells *via* Suppressing the Polarization of M2 Macrophages

**FIGURE 6:**
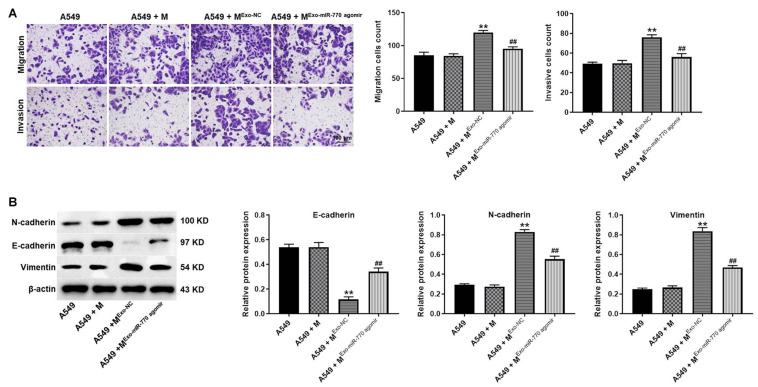
Exosomal miR-770 significantly inhibited the migration, invasion and EMT process of NSCLC cells *via* suppressing the polarization of M2 macrophages. **(A)** A549 cells were co-cultured with macrophages, M^*exo–NC*^ or M^*Exo–mIR–770 agomir*^. Then, the migration and invasion of NSCLC cells was tested by transwell assay. **(B)** The protein levels of E-cadherin, N-cadherin and vimentin in A549 cells were detected by western blot. The relative expressions were quantified by normalizing to β-actin. *N* = 3; **P < 0.01 compared to A549 cells, ^##^*P* < 0.01 compared to A549 + M^*exo–NC*^.

In order to investigate whether exosomal miR-770 could inhibit the migration and invasion of NSCLC cells by inhibiting M2 macrophages polarization, transwell assay was performed. As indicated in Figure 6A, A549 cells after co-culturing with M2 macrophages induced by A549/agomir-NC-exo exhibited increased cell migration and invasion abilities; however, this phenomenon was notably reversed by exosomes containing miR-770 agomir. Meanwhile, exosomes with upregulated miR-770 significantly reversed the effect of macrophages incubated with A549/agomir-NC-exo on vimentin, N-cadherin and E-cadherin levels (Figure 6B). Taken together, exosomal miR-770 could inhibit the migration, invasion and EMT process of NSCLC cells *via* suppressing the polarization of M2 macrophages.

### MAP3K1 Was the Direct Target of miR-770

**FIGURE 7:**
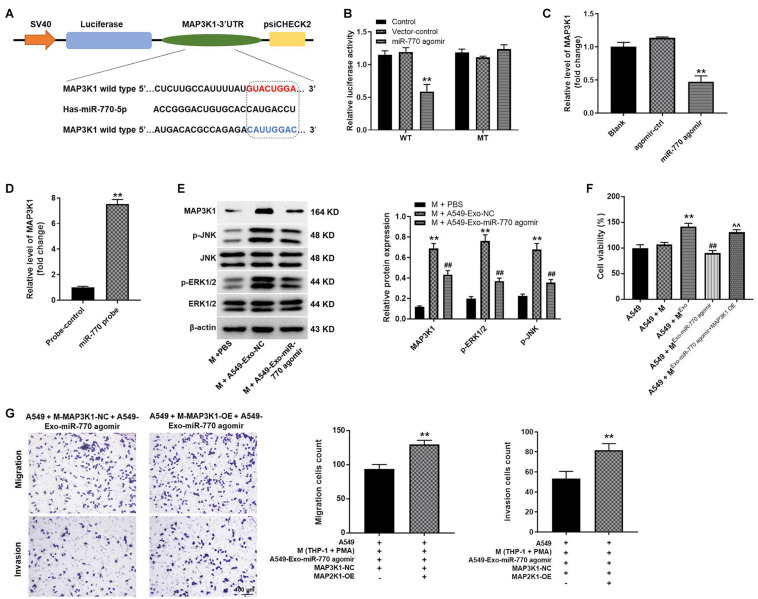
MAP3K1 was found to be the direct target of miR-770. **(A)** The potential target of miR-770 was predicted by targetscan. **(B)** The relative luciferase activity was detected by dual luciferase reporter assay. **(C)** Macrophages were transfected with agomir-ctrl or miR-770 agomir. Then, the level of MAP3K1 in macrophages was detected by RT-qPCR. **(D)** The correlation between miR-770 and MAP3K1 was tested by RNA pull-down. **(E)** Macrophages were co-cultured with A549-Exo-NC or A549-Exo-miR-770 agomir. The protein levels of MAP3K1, p-ERK1/2, ERK1/2, JNK or p-JNK in macrophages were detected by western blot. The relative expressions were quantified by normalizing to β-actin. **(F)** The viability of NSCLC cells was tested by CCK-8 assay. **(G)** The migration and invasion of NSCLC cells was tested by transwell assay. *N* = 3; ^∗∗^*P* < 0.01 compared to M + PBS or A549 + M-MAP3K1-NC + A549-Exo-miR-770 agomir, ^##^*P* < 0.01 compared to M + A549-Exo-NC.

To find the target mRNA of miR-770, targetscan (version 7.2^2^) was used. As indicated in Figure 7A, MAP3K1 might be the potential target of miR-770, and the relative luciferase activity of reporter vectors containing WT-MAP3K1 was significantly inhibited by miR-770 agomir (Figure 7B). However, miR-770 agomir had very limited effect on luciferase activity of reporter vectors containing MT-MAP3K1 (Figure 7B). Meanwhile, the level of MAP3K1 in macrophages was significantly downregulated by miR-770 overexpression (Figure 7C), and the enrichment of MAP3K1 was significantly upregulated by miR-770-probe, compared with probe-control (Figure 7D). In addition, the levels of MAP3K1, p-JNK, and p-ERK1/2 were significantly increased in macrophages incubated with A549/agomir-ctrl-exo; however, this phenomenon was reversed when macrophages were incubated with A549/miR-770 agomir-exo (Figure 7E). Furthermore, macrophages co-treated with A549/miR-770 agomir-exo and MAP3K1-OE notably increased the viability, migration and invasion of A549 cells, compared to macrophages treated with A549/miR-770 agomir-exo (Figures 7F,G). Taken together, exosomal miR-770 could inhibit the polarization of M2 macrophages *via* inhibition of MAPK signaling.

### Exosomal miR-770 Significantly Inhibited the Tumor Growth of NSCLC *in vivo via* Suppressing the M2 Polarization of Macrophages

**FIGURE 8:**
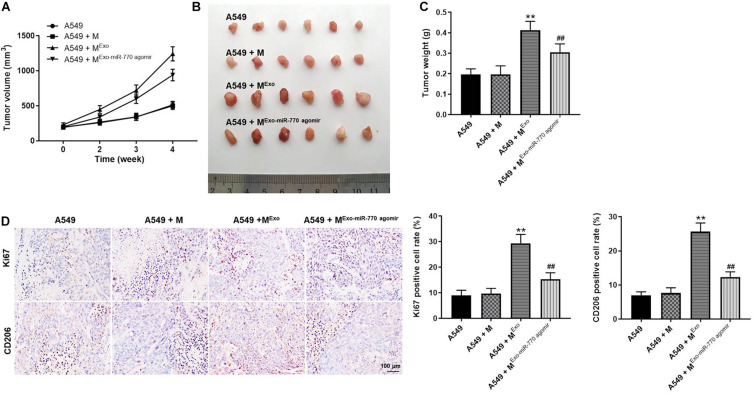
Exosomal miR-770 significantly inhibited the tumor growth of NSCLC *in vivo via* suppressing the M2 polarization of macrophages. **(A)** The tumor volume of mice was measured every week for four weeks. **(B)** The tumor tissues of mice were pictured. **(C)** Tumor weights of mice were measured. **(D)** The expressions of Ki67 and CD206 in tissues of mice were detected by IHC staining. The relative expressions were calculated. *N* = 6; **P < 0.01 compared to A549 cells, ^##^*P* < 0.01 compared to A549 + M^*Exo*^.

To investigate the function of exosomal miR-770 in NSCLC *in vivo*, xenograft mice model was established. As revealed in Figures 8A–C, A549-exo-treated macrophages significantly increased the tumor volume and tumor weight of mice, while macrophages treated with A549/miR-770 agomir-exo significantly reversed these phenomena. Meanwhile, the positive rate of Ki67 and CD206 staining in tumor tissues was notably downregulated in A549 + M^*Exo–miR–770 agomir*^ group, compared with the A549 + M^*Exo*^ group (Figure 8D). To sum up, exosomal miR-770 could inhibit the tumor growth of NSCLC *in vivo via* suppressing the M2 polarization of macrophages.

### Exosomal miR-770 Significantly Induced the Apoptosis in Tumor Tissues of Mice

**FIGURE 9:**
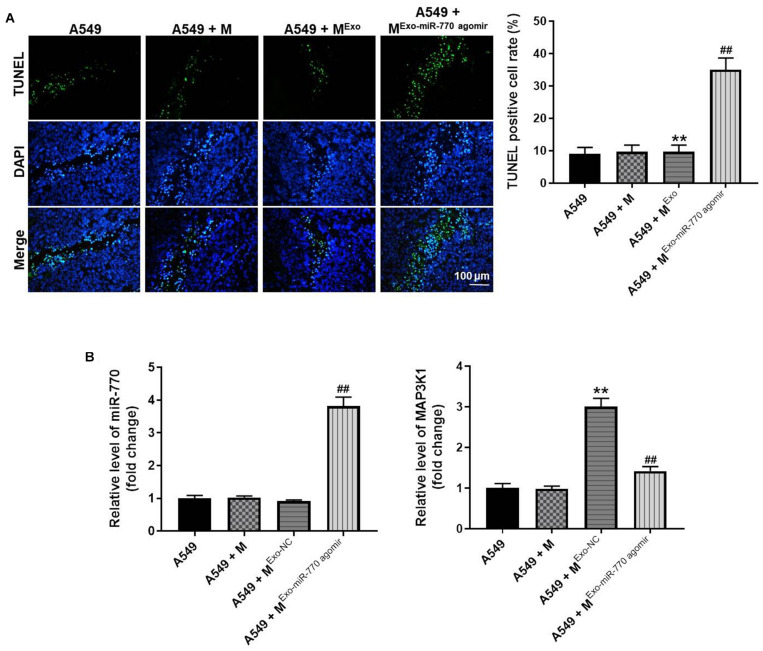
Exosomal miR-770 significantly induced the apoptosis in tumor tissues of mice *via* suppressing the M2 polarization of macrophages. **(A)** The apoptosis in tumor tissues was detected by TUNEL staining. **(B)** The levels of miR-770 and MAP3K1 in tumor tissues of mice were investigated by RT-qPCR. *N* = 6; **P < 0.01 compared to A549 cells, ^##^*P* < 0.01 compared to A549 + M^*Exo*^.

In order to further investigate the effect of exosomal miR-770 on tumor growth of NSCLC, TUNEL staining was performed. The data showed that the positive rate of TUNEL staining was significantly increased in macrophages treated with A549/miR-770 agomir-exo, compared with the A549 + M^*Exo*^ group (Figure 9A). Consistently, the level of miR-770 was notably upregulated in macrophages treated with A549/miR-770 agomir-exo, compared with the A549 + M^*Exo*^ group (Figure 9B). Meanwhile, A549-exo-treated macrophages significantly increased the level of MAP3K1 in tumor tissues of mice, while this phenomenon was significantly reversed by macrophages treated with A549/miR-770 agomir-exo (Figure 9B). In summary, Exosomal miR-770 significantly induced the apoptosis in tumor tissues of mice.

## Discussion

Exosomes are secreted by many types of cells (especially tumor cells), and they have been reported to be closely correlated with cancer progression (Zeng and Fu, 2020; Wang et al., 2021). Previous studies revealed that exosomes could construct a microenvironment which can induce the cancer progression *via* the association between cancer cells and surrounding stromal cells (Yeon et al., 2019; Chen C. et al., 2020). Meanwhile, M2 macrophages are known to play crucial roles in tumor development as they can induce tumor metastasis and recurrence (Biswas et al., 2019; Cheng et al., 2019). In the current research, exosomes derived from tumor cells promoted the M2 polarization of macrophages, which in turn increased the invasion, migration and EMT of NSCLC cells. Our data suggest that tumor cell-derived exosomes are crucial members of the tumor microenvironment and act as an important regulator in the cross-talk between different cells.

It has been reported that dysregulation of miRNAs often leads to cancer occurrence (Geng et al., 2018; Park et al., 2018). MiR-770 is known to have important biological functions in cancer tumorigenesis and metastasis (Feng et al., 2019; Jia et al., 2020). Our current study found that miR-770 was significantly downregulated in exosomes, and it could suppress the migration and invasion of NSCLC cells. Meanwhile, the overexpression of miR-770 in exosomes impaired exosome secretion and reversed the polarization of M2 macrophages, thus suppressing the migration, invasion and EMT process of NSCLC cells. All these data suggested that NSCLC cell–derived exosomal miR-770 could play a key role in the tumor microenvironment and is crucial for tumor metastasis. A previous report indicated that miR-770 could inhibit the EMT process of NSCLC *via* regulation of Wnt/β-catenin signaling (Zhang et al., 2017). Our study was similar to this previous research. β-catenin is known to be positively correlated with EMT process (Li et al., 2021; Yiu et al., 2021). Therefore, the similar function between Wnt/β-catenin and tumor cell-derived exosomes might result in this similarity.

It has been confirmed that the MAPK/ERK1/2 signaling pathway is involved in the process of tumor cell growth, migration and invasion (Haupt et al., 2020; Li et al., 2020a). Some data have revealed that exosomes could activate phosphorylation of cell growth-related pathways (including MAPK/ERK1/2) (Qu et al., 2020; Zhang et al., 2020). In our study, MAP3K1 was found to be the target mRNA of miR-770. In addition, NSCLC-exos can induce phosphorylation of ERK, and this phenomenon was found to be partially inhibited by exosomal miR-770. This result suggested that the cell growth-related signals in macrophages can be activated by exosomes to increase tumor cell proliferation. Meanwhile, our finding also found that NSCLC-exos-induced NSCLC cell growth was reversed due to exosomal miR-770. Based on these data, these results revealed that the MAPK/ERK1/2 signaling pathway might play an important role in tumor microenvironment.

Zhang et al. (2017) found that miR-770 could inhibit the tumorigenesis and EMT by targeting JMJD6 and regulating WNT/beta-catenin pathway in non-small cell lung cancer. Meanwhile, our study revealed that exosomal miR-770 could inhibit the tumorigenesis and EMT process of NSCLC through targeting MAP3K1. Thus, our study further explored the function of tumor cell-derived exosomal miR-770 in NSCLC. In addition, JMJD6 could be targeted by miR-770, and WNT/β-catenin pathway was positively regulated by JMJD6 (Zhang et al., 2017). Moreover, β-catenin was reported to be negatively correlated with E-cadherin (negative mediator in EMT process) (Teeuwssen and Fodde, 2019). On the other hand, we used targetscan database and found that MAP3K1 was the target of miR-770. Thereby, our study indicated that miR-770 could regulate the tumorigenesis of NSCLC through targeting other mRNAs. Meanwhile, Yongmei Chang et al. (2017) found that activation of MAP3K1 and JNK signaling could promote the EMT process. Our study was consistent to Yongmei Chang et al., suggesting that tumor cell-derived exosomal miR-770 inhibits the tumorigenesis and EMT process of NSCLC through targeting MAP3K1.

Of course, there are some shortcomings in this research as follows: (1) rescue experiments need to be performed to further verify the role of MAPK signaling in exosomes-mediated tumor cell growth; (2) more mRNAs targeted by miR-770 are needed to be explored; (3) the function of MAP3K1 in NSCLC *in vivo* remains unclear. Thereby, more investigations are needed in the coming future.

In conclusion, tumor cell-derived exosomal miR-770 inhibits M2 macrophage polarization *via* targeting MAP3K1. Therefore, our study shed new lights on exploring the new strategies for the treatment of NSCLC.

## Data Availability Statement

The original contributions presented in the study are included in the article/Supplementary Material, further inquiries can be directed to the corresponding author/s.

## Ethics Statement

The animal study was reviewed and approved by Ethical Committee of Peking University Shenzhen Hospital.

## Author Contributions

XL and GM conceived and supervised the study. JL, RL, and JW designed the experiments. XL, DW, HC, and QH performed the experiments. All authors reviewed the results and approved the final version of the manuscript.

## Conflict of Interest

The authors declare that the research was conducted in the absence of any commercial or financial relationships that could be construed as a potential conflict of interest.

## Footnotes

1 https://cancergenome.nih.gov/publications/publicationguidelines

2 http://www.targetscan.org/vert_72/

## Supplementary Material

Supplementary Figure 1 MiR-770 agomir significantly inhibited the proliferation of NCI-H1650 cells *via* inducing the apoptosis. NCI-H1650 cells were transfected with agomir-ctrl or miR-770 agomir. **(A)** The viability of NCI-H1650 cells was tested by CCK-8 assay. **(B)** The proliferation of NCI-H1650 cells was tested by Ki-67 staining. **(C)** The apoptosis of NCI-H1650 cells was investigated by flow cytometry. *N* = 3; ^∗∗^*P* < 0.05 compared to control.
