# Supplementary figures and images for: Tumor Cell-Derived Exosomal miR-770 Inhibits M2 Macrophage Polarization via Targeting MAP3K1 to Inhibit the Invasion of Non-small Cell Lung Cancer Cells
Source: Front Cell Dev Biol. 2021 Jun 14;9:679658. doi: 10.3389/fcell.2021.679658 (PMC8236888; doi:10.3389/fcell.2021.679658)

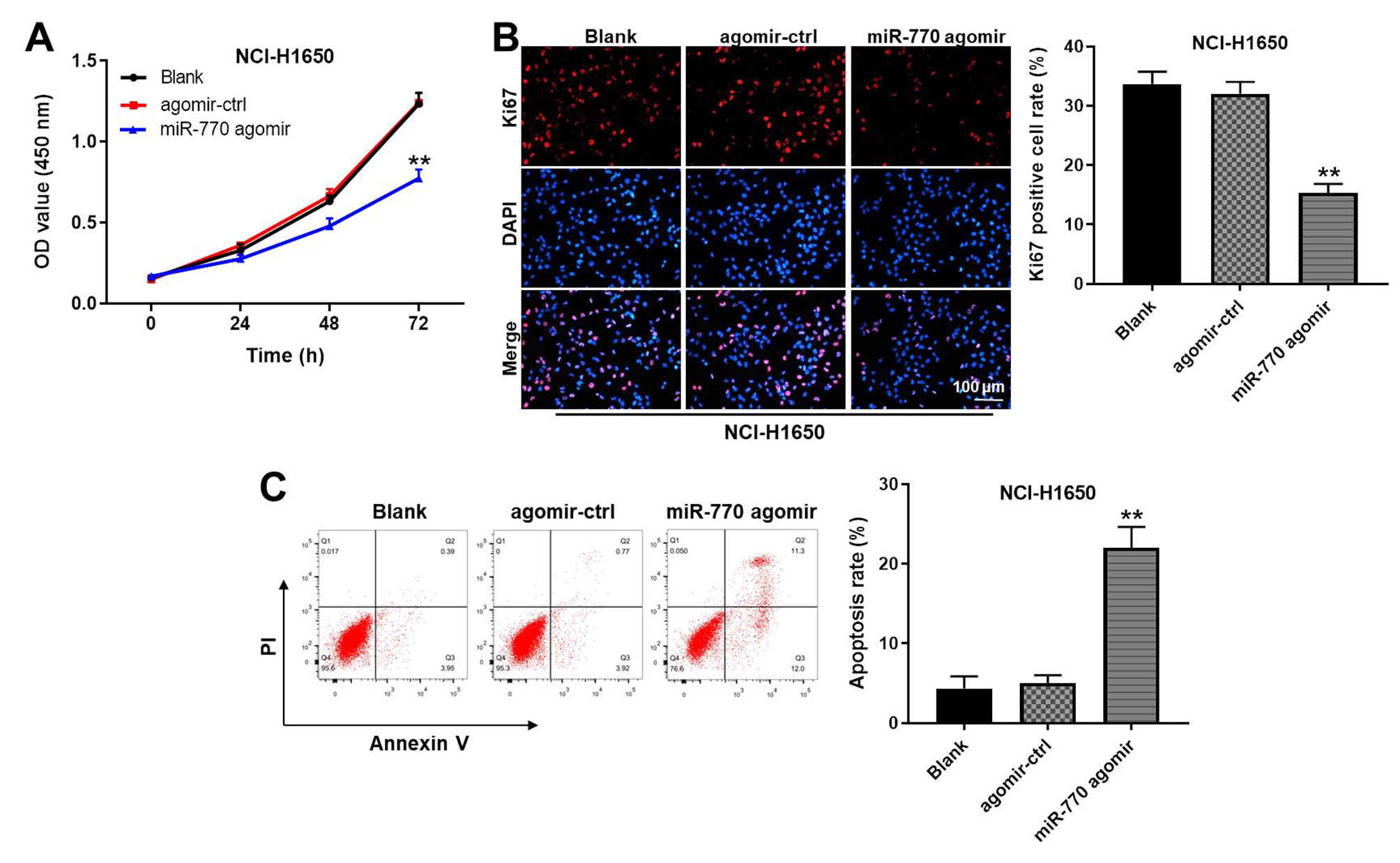

Supplement: Supplementary Figure 1 — MiR-770 agomir significantly inhibited the proliferation of NCI-H1650 cells via inducing the apoptosis. NCI-H1650 cells were transfected with agomir-ctrl or miR-770 agomir. (A) The viability of NCI-H1650 cells was tested by CCK-8 assay. (B) The proliferation of NCI-H1650 cells was tested by Ki-67 staining. (C) The apoptosis of NCI-H1650 cells was investigated by flow cytometry. N = 3; ∗∗P < 0.05 compared to control. [file Image_1.JPEG]
